# Altered intercellular communication in lung fibroblast cultures from patients with idiopathic pulmonary fibrosis

**DOI:** 10.1186/1465-9921-7-122

**Published:** 2006-09-27

**Authors:** Angela Trovato-Salinaro, Elisa Trovato-Salinaro, Marco Failla, Claudio Mastruzzo, Valerio Tomaselli, Elisa Gili, Nunzio Crimi, Daniele Filippo Condorelli, Carlo Vancheri

**Affiliations:** 1 Department of Internal Medicine and Specialistic Medicine, Section of Respiratory Diseases University of Catania, Catania, Italy; 2 Department of Chemical Sciences, Section of Biochemistry and Molecular Biology, University of Catania, Catania, Italy

## Abstract

**Rationale:**

Gap junctions are membrane channels formed by an array of connexins which links adjacent cells realizing an electro- metabolic synapse. Connexin-mediated communication is crucial in the regulation of cell growth, differentiation, and development. The activation and proliferation of phenotypically altered fibroblasts are central events in the pathogenesis of idiopathic pulmonary fibrosis. We sought to evaluate the role of connexin-43, the most abundant gap-junction subunit in the human lung, in the pathogenesis of this condition.

**Methods:**

We investigated the transcription and protein expression of connexin-43 and the gap-junctional intercellular communication (GJIC) in 5 primary lung fibroblast lines derived from normal subjects (NF) and from 3 histologically proven IPF patients (FF).

**Results:**

Here we show that connexin-43 mRNA was significantly reduced in FF as demonstrated by standard and quantitative RT-PCR. GJIC was functionally evaluated by means of flow-cytometry. In order to demonstrate that dye spreading was taking place through gap junctions, we used carbenoxolone as a pharmacological gap-junction blocker. Carbenoxolone specifically blocked GJIC in our system in a concentration dependent manner. FF showed a significantly reduced homologous GJIC compared to NF. Similarly, GJIC was significantly impaired in FF when a heterologous NF line was used as dye donor, suggesting a complete defect in GJIC of FF.

**Conclusion:**

These results suggest a novel alteration in primary lung fibroblasts from IPF patients. The reduced Cx43 expression and the associated alteration in cell-to-cell communication may justify some of the known pathological characteristic of this devastating disease that still represents a challenge to the medical practice.

## Background

Idiopathic pulmonary fibrosis (IPF) is the most common among interstitial pneumonias of unknown origin and one of the most aggressive interstitial lung diseases. Although the pathogenesis is incompletely understood, the activation and proliferation of lung fibroblasts which lead to excessive extracellular matrix components (ECM) accumulation and altered mesenchymal cell interactions, are believed to be critical events driving the chronic and progressive course of IPF [1].

The presence of aggregates of actively proliferating fibroblasts termed "fibroblast foci" is a hallmark of usual interstitial pneumonia (UIP) in IPF [2,3]. It has been suggested that abnormal interaction between parenchymal fibroblasts may set in motion a series of cellular events and matrix alterations which result in altered mesenchymal cell phenotype and fibrogenesis [4].

Gap junctions are specialized membrane regions composed of aggregates of transmembrane channels that directly connect the cytoplasm of adjacent cells [5-7]. The passage of ions and small molecular weight molecules through gap junction channels results in metabolic and electrical coupling of cells thus allowing rapid intercellular communication and synchronization of cell activities. Gap junction intercellular communication (GJIC) is believed to play a critical role in cell proliferation, tissue differentiation and homeostasis [8]. Gap junctions are formed by the conjunction of two hemichannels called connexons, each composed of an hexameric assembly of subunit proteins called connexins [9]. Connexins are encoded by a large multigene family. In mammals 20 different members of this gene family are known [10,11]. Connexin 43 (Cx43) was one of the first connexins discovered in fibroblasts [12], and one of the most abundant in human lung fibroblasts [13]. Gap junctions in the lung are important in the regulation of cell proliferation, differentiation and development [14,15], furthermore, recent observations suggest that a down-regulation of GJIC might play a relevant role in lung cancer [16-18]. Similarly, early Cx43 down-regulation during wound repair has been shown. This phenomenon correlates not only with the rapidity and efficiency of wound closure but also with crucial events in wound repair such as inflammatory cell recruitment, structural cell proliferation and migration [19]. A similar inverse relationship between connexin expression and cellular proliferation has been described in hyper-proliferative skin diseases like psoriasis [20,21].

Uncontrolled fibroblast proliferation and altered fibroblast phenotypes are considered crucial events in the onset and evolution of IPF. Nevertheless, to date, the role of GJIC in the pathogenesis of IPF has not been investigated.

In the present study the expression of Cx43 in normal fibroblasts and in fibrotic fibroblasts from patients with IPF/UIP was studied. Moreover, to better understand the role played by gap junctions in the pathogenesis of IPF, we functionally evaluated the gap junctional intercellular communication in normal and fibrotic fibroblasts by measuring gap-junctional coupling using flow cytometry.

## Methods

### Primary lung fibroblast cultures

Primary lines of normal human lung fibroblasts were established by using an outgrowth from explant following the method described by Jordana et al. [22]. Five normal fibroblast lines were derived from histologically normal areas of lung specimens from 5 patients undergoing resective surgery for cancer. Their ages ranged from 52 to 61 yr. Three fibrotic lung fibroblast lines were established from histologically proven fibrotic lung tissue of 3 patients with idiopathic pulmonary fibrosis undergoing surgical lung biopsy for diagnostic means. Their ages ranged from 45 to 55 years. The Local Ethic Committee gave its approval for the study and all of the patients gave their written informed consent. In all experiments, cultured fibroblasts were used at a passage earlier than the fifth.

### RT-PCR assays for connexin transcripts

Total RNA extraction and cDNA synthesis were performed as previously described [23]. The following specific fragments were amplified:

1) Cx26 mRNA (accession number NM_004004): a 290-bp fragment, encompassing nucleotides 146–435, was amplified using the following primers: forward primer: 5'-TTCCTCCCGACGCAGAGCAA-3'; reverse primer: 5'-ACACGAAGATCAGCTGCAGG-3'. Human liver RNA (Ambion Inc., Austin, Texas, USA) was used as positive control sample.

2) Cx32 mRNA (Accession number NM_000166): a 221-bp fragment, encompassing nucleotides 26–246, was amplified using the following primers: forward primer: 5'-AGGTGTGGCAGTGACAGGGA-3'; reverse primer: 5'-TGTTGCAGCCAGGCTGGAGT-3'. Human liver RNA (Ambion Inc.) was used as positive control sample.

3) Cx43 mRNA (Accession number NM_001101): a 336-bp fragment, encompassing nucleotides 158–493, was amplified using the following primers: forward primer: 5'-ACTTGGCGTGACTTCACTAC-3'; reverse primer: 5'-CATGAGCCAGGTACAAGAGT-3'. Human heart RNA (Ambion Inc.) was used as positive control sample.

### Quantitative real-time RT-PCR

Real-time quantitative RT-PCR experiments were performed in the ABI Prism 7700 System (Applied Biosystems, Foster City, Calif., USA). The following oligonucleotides were used: – forward primer: 5'-TTCATTTTACTTCATCCTCCAAGGA-3', – reverse primer: 5'-CAGTTGAGTAGGCTTGAACCTTGTC-3', – fluorogenic probe: 5' FAM-ACTTGGCGTGACTTCA-TAMRA 3'. Commercially available normal human lung, kidney, heart RNA (Ambion Inc.) and cerebral cortex RNA, extracted from an autoptic sample as previously described [24], were also analysed as positive controls. Relative quantification of Cx mRNAs was performed by the 2^-ΔΔCt ^method previously decribed [25].

### Western blots

Cells grown in 100 mm culture dishes of five normal and three fibrotic lung fibroblast lines were washed with PBS buffer and were homogenized in ice-cold 40 mM Tris-HCl buffer, pH 7.4, containing 2.5% SDS detergent, 1 mM phenylmethylsulfonyl fluoride (PMSF), and a cocktail of protease inhibitors diluted at 1:200 (Sigma-Aldrich P8340). The homogenization medium was further supplemented with the phosphatase inhibitors sodium orthovanadate and sodium fluoride at 1 and 10 mM concentration, respectively. After homogenization, samples were sonicated for 30 sec. Protein concentration was determined with the bicinchoninic acid method, using BSA as the standard. Samples were separated on 10% polyacrylamide gels. Before being loaded onto gels, samples were boiled in sample buffer (40 mM Tris-HCl buffer, pH 7.4, containing 2.5% SDS, 5% 2-mercaptoethanol, 5% glycerol, 0.025 mg/ml bromophenol blue) for 4 min. Resolved proteins were transferred to nitrocellulose membrane (0.45 μm) (BIO-RAD Hercules, CA, USA) in transfer buffer [25 mM Tris, 192 mM glycine, and 20% (v/v) methanol] containing 0.05% SDS. Membrane was blocked for 2 hr at 22°C in 20 mM Tris, pH 7.4, 150 mM NaCl, and 0.1% Tween 20 (TBS-T) containing 3% BSA and incubated with primary antibody overnight at 4°C in TBS-T containing 1% BSA. Blot was tested with a monoclonal mouse Cx43 antibody diluted at 1:250 (Chemicon Inc Temecula, CA, USA). After incubation with primary antibody, membrane was washed in TBS-T and then incubated for 1 hour at room temperature in TBS-T containing 1% BSA and incubated with anti-mouse horseradish peroxidase-conjugated secondary antibody at dilution of 1:10000. Blot was washed in TBS-T and then incubated for 3 min using the SuperSignal chemiluminescence detection Kit system (Pierce Chemical Co, Rockford, USA).

Separate blots, loaded with the same samples, were also incubated with the mouse anti-β-actin monoclonal antibody (Sigma-Aldrich A4700, 1:300 dilution) as a control for the quality of the protein preparations. Specific band was visualized by using the SuperSignal chemiluminescent detection system (Pierce Chemical Co, Rockford, USA).

### Dye coupling by flow cytometry

Functional assay of the gap junctional activity was assessed with a dye-loading technique by means of flow cytometry [26]. Donor cells were loaded with calcein acetoxymethyl ester (CAL) (Molecular Probes, Eugene, OR) and 1,1' dioctadecyl-3,3,3',3'-tetramethylindocarbocyanine perchlorate (DiI) (Molecular Probes). Donor cells were trypsinized and added to a monolayer culture of unstained recipient cells (CAL-DiI-) at a ratio of 1:5. After different incubation times these cocultures were analyzed by FACS.

Cocultured experiments were performed either using the same cell line as donor and recipient cells (homologous coupling) either using different cell lines as donor and recipient cells (heterologous coupling). Carbenoxolone (CBX) (Sigma, St. Louis, Missouri, USA), a specific gap junction blocker, was used to ascertain whether the intercellular transfer assay described above was dependent on intercellular gap junction communication.

### Statistical analysis

Results are shown as means ± standard deviation (SD). GJIC assay results represent the number of calcein-positive recipient cells (CAL+DiI-) expressed as percentage of total recipient cells (DiI-). Comparisons between groups were made by means of two way ANOVA or Student's unpaired t-test where appropriate; P values of 0.05 or less were considered to be statistically significant.

## Results

### Cx43, Cx32, Cx26 mRNAs in cultured human lung fibroblasts

In order to establish whether, and identify which connexins were expressed in cultured lung fibroblasts, specific RT-PCR amplifications were performed and the products were separated by agarose-gel electrophoresis.

We established and used 5 fibroblast cell lines derived from histologically normal areas of surgical specimens from 5 patients undergoing to resective lung surgery for cancer and 3 fibrotic fibroblast cell lines derived from surgical biopsies of 3 different patients with histologically proven UIP/IPF.

**Figure 1:**
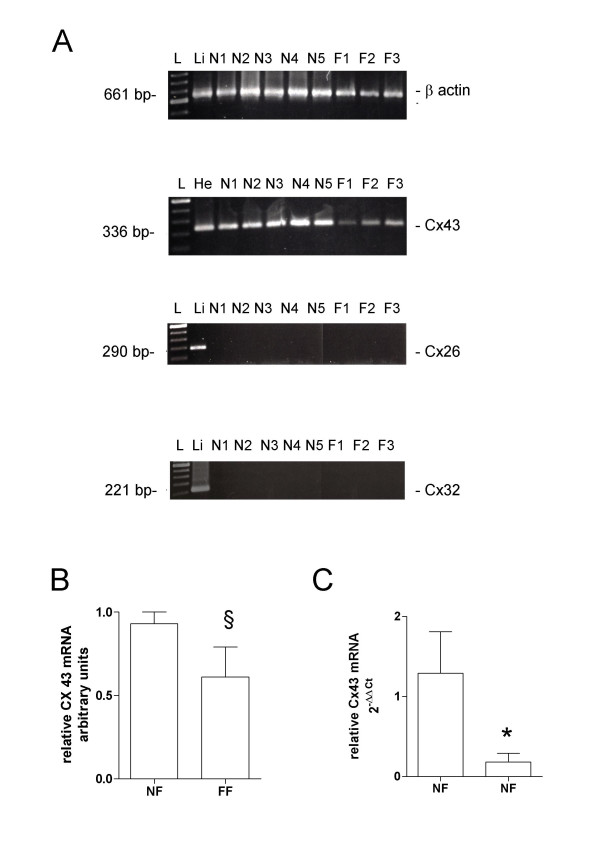
(**A**) RT-PCR analysis of human Cx43, Cx26, Cx32 mRNA expression in cultured lung fibroblasts. Representative results obtained by analysis of RNA extracted from 5 normal lung fibroblast cultures (N1-5) and 3 fibrotic fibroblast cultures (F1-3). (B) densitometry analysis of RT-PCR analysis of human Cx43 mRNA expression in cultured lung fibroblasts. RNA samples from human liver (Li) and heart (He) were also analyzed as positive controls. L: 100-bp ladder. (C) Human Cx43 mRNA level measured by quantitative real-time RT-PCR in cultured normal and fibrotic fibroblasts. Results obtained from commercially available human lung RNA sample has been reported as control. Relative quantification was performed by the 2^-ΔΔCt ^method using as calibrator the value obtained from human normal lung. Data represent mean ± SD of 5 primary normal fibroblast lines and 3 fibrotic fibroblast lines. NF normal lung fibroblast, FF fibrotic lung fibroblast. §p = NS, * p < 0.05 in comparison to normal fibroblasts.

Transcripts for Cx32 and Cx26 were absent in both normal and fibrotic fibroblasts, while Cx43 mRNA was intensely expressed in all the samples analyzed (Fig. 1A). Although a trend toward lower levels of Cx43 mRNA in fibrotic fibroblasts in comparison to normal lung fibroblasts was observed (Fig. 1B)., the poor reliability of conventional RT-PCR as a quantitative method did not allow any final conclusion. Therefore, we repeated the Cx43 mRNA analysis using a real-time quantitative RT-PCR based on the Taqman method. Results obtained with a panel of human tissues were in agreement with the profile of expression of Cx43 reported in literature [27,28], thus confirming the specificity of the method: heart showed the highest level, while Cx43 expression was undetectable in liver tissue and intermediate values were observed in lung, kidney and cerebral cortex (data not shown).

Connexin 43 mRNA expression in normal fibroblasts was in perfect agreement with that of a commercially available human lung mRNA sample. However, Cx43 mRNA expression in fibrotic cell lines of different patients was significantly reduced when compared to normal fibroblast cell lines (Fig. 1C, 12-fold reduction, p < 0.05).

### Cx43 protein in cultured human lung fibroblasts

**Figure 2:**
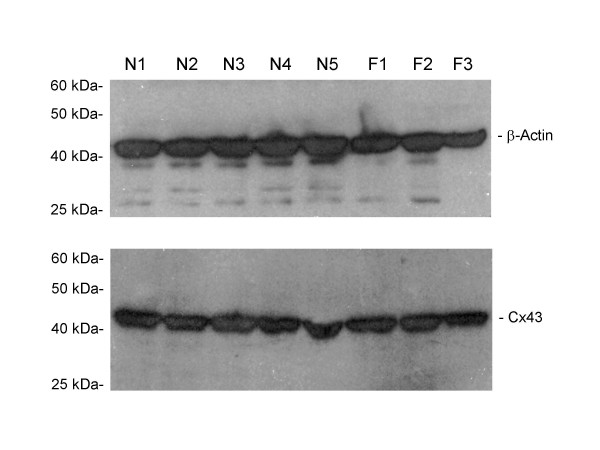
Western blots for detection of Cx43 and β-actin in normal lung fibroblast cultures and fibrotic fibroblast cultures.

In order to establish if the observed decrease of Cx43 mRNA levels in fibrotic cell lines is paralleled by a decrease of the corresponding protein, the level of Cx43 protein was determined by western blot assays. As shown in fig. 2, no difference in the amount of Cx43 protein was detected between normal and fibrotic fibroblasts.

### Functional assay of gap junctional intercellular communication

Functional assay of the gap junctional activity was assessed with a dye-loading technique by means of flow cytometry [29]. Lipophilic Dil stains plasma membranes of cells with a red fluorescence, while the membrane-permeable calcein-AM is intracellularly hydrolyzed by non-specific esterases producing the green fluorescent polyanionic calcein. In order to study the dye spreading through adjacent cells, donor cells are double stained with Dil and calcein-AM and then seeded onto a monolayer of unstained recipient cells with a fixed donor to recipient ratio of 1:5. The intercellular passage of calcein through gap junction channels results in the progressive increase of green fluorescent recipient cells.

**Figure 3:**
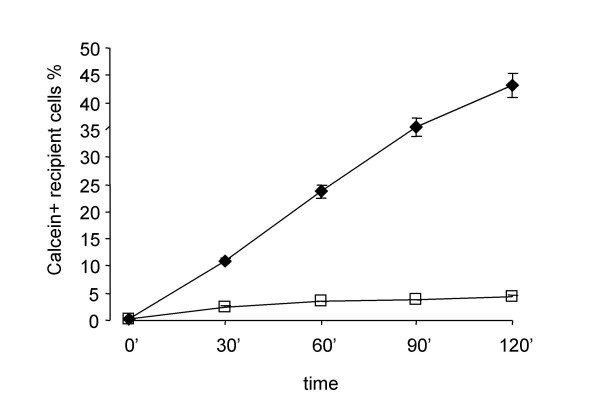
Time course of homologous gap junctional intercellular communication (GJIC) and effect of carbenoxolone on the calcein dye transfer of normal human lung fibroblasts. Data represent the number of calcein-positive recipient cells as percentage of total recipient cells and are the averages of four different normal lines ± SD. ◆ Normal fibroblasts, □ Normal fibroblasts treated with 100 μM carbenoxolone.

**Figure 4:**
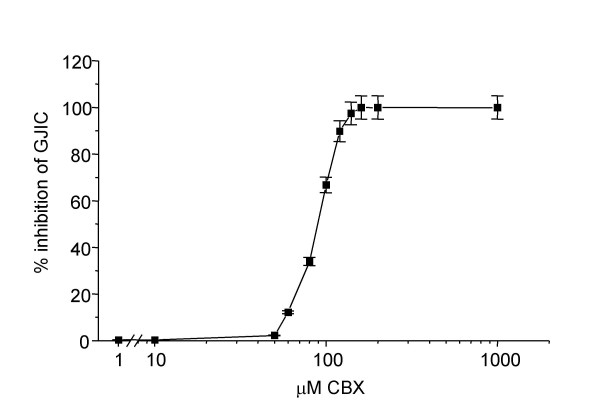
Dose-response curve of CBX on gap junctional intercellular communication in normal human lung fibroblasts. Data are expressed as the percentage of maximal inhibition of calcein dye transfer and are the averages of four dishes ± SD.

**Figure 5:**
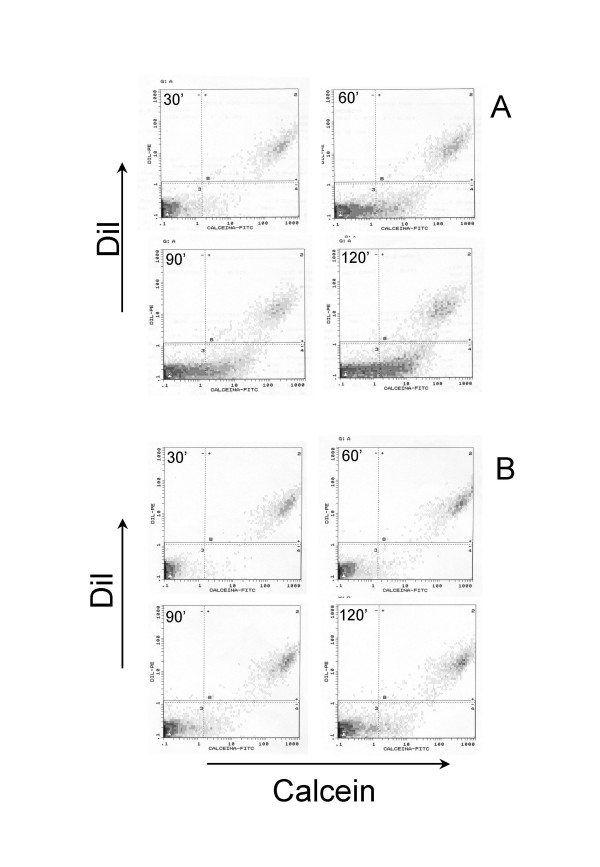
Representative flow cytometry dot plots showing calcein dye transfer trough gap junctions at different time points in a normal fibroblast line in the absence (A) and in the presence (B) of CBX (100 uM). At time 0 the recipient cells (DiI and calcein negative) are in the left-bottom box and donor cells (DiI and calcein positive) in the right-upper box. Note in A the time-dependent increase in calcein positive cells in the recipient cell populations (right-bottom box). This increase is almost completely blocked by CBX in B.

The number of calcein-labeled recipient cells increased rapidly during the first two hours after the initial contact between donor and recipient cells (Fig. 3). In order to show that intercellular spreading of calcein was taking place by flow through gap junction channels, the assay was performed in the presence and in the absence of carbenoxolone (CBX), a pharmacological gap junction blocker. CBX is a moderately lipophilic glycyrrhetinic acid derivative that has been shown to act directly on gap junctions to reduce conductance by up to 80%, although the exact mechanism underlying this effect remains to be determined [30-32]. As expected, CBX specifically blocked GJIC in our system in a concentration dependent manner as shown in Figs. 3, 4 and 5A.

### Calcein dye coupling in normal and fibrotic fibroblasts

**Figure 6:**
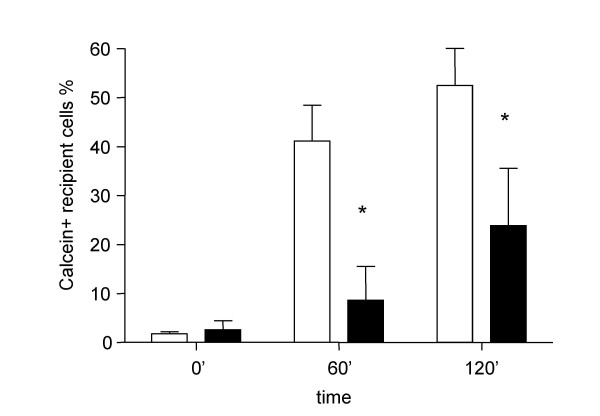
Homologous GJIC assayed by means of the calcein dye transfer technique on normal and fibrotic human lung fibroblast at different time points. Open bar represents normal human lung fibroblasts, N = 5; Filled bar represents fibrotic human lung fibroblasts N = 3; All the experiments were conducted in triplicate * p < 0.01 vs. normal fibroblasts.

Using the calcein dye transfer assay described above we studied gap junctional intercellular communication in our normal and fibrotic fibroblast cell cultures. In normal fibroblasts calcein spreading occurred rapidly. After 2 hours more than 50% of the cells were stained (Fig. 6). At 0, 60 and 120 min, the percentage of Calcein+Dil- recipient fibroblasts was respectively 1.7% ± 0.4, 41.1% ± 7.4 and 52.4% ± 7.8 (mean% ± SD), respectively. Primary fibroblast cultures derived from UIP/IPF biopsies showed a significantly reduced GJIC as underlined by slowed calcein spreading. At the same time points described above, recipient Calcein+Dil- cells were 2.6% ± 1.8, 8.6% ± 6.9 (p < 0.01 vs. normal fibroblasts) and 23.8% ± 11.7 (p < 0.01 vs. normal fibroblasts), respectively.

When the number of donor cells seeded onto the recipient monolayer with the number attached after 30 minutes of incubation were compared, no significant differences between normal and fibrotic fibrobasts were detected (data not shown). This excludes the possibility that gross abnormalities in cell adhesion are responsible for the observed differences.

In the experiments described thus far donor and recipient cells belonged to the same cell line (homologous GJIC). In order to exclude the possibility that differences in loading and retention of intracellular marker dye calcein in donor cells might account for the observed differences between fibrotic and normal fibroblasts, we performed experiments using a single normal fibroblast cell line as donor cells for both fibrotic and normal recipient cell lines (heterologous GJIC).

**Figure 7:**
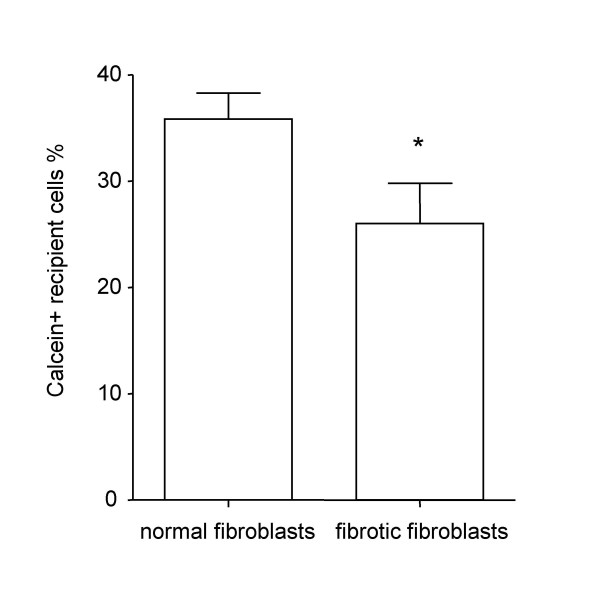
Heterologous GJIC in normal and fibrotic human lung fibroblasts. A single normal fibroblast cell line was used as dye donor over all of the other recipient cell lines. Normal fibroblasts (N = 5) vs. fibrotic fibroblasts (N = 3) at 120 minutes. All the experiments were conducted in triplicate. * p < 0.05.

In confirmation of our homologous GJIC experiments, heterologous GJIC was significantly reduced at 120 minutes in fibrotic fibroblasts when compared to normal recipient fibroblast lines (26% ± 8.1 vs. 35.6% ± 1.5, p < 0.05) (Fig. 7).

## Discussion

The pathogenesis of IPF is still unclear. Nevertheless, the presence and the extent of fibroblastic foci in the lung of affected patients is one of the most prominent features associated with IPF progression and survival [33-35]. The presence of activated myofibroblasts within fibroblastic foci suggests that an unknown inciting factor, possibly acting in concert with a genetic predisposition or an epigenetic alteration, might underlie the uncontrolled proliferation of fibroblasts and ECM deposition, leading to lung fibrosis [36].

In this context, it is believed that abnormal cell-to-cell communication between parenchymal cells may lead to the emergence of altered fibroblast phenotypes and uncontrolled fibroblast proliferation. Among the structures that maintain cell contact, gap junctions are believed to be the main players in cell-to-cell communication, by allowing the sharing of small cytoplasmic molecules and ions between adjacent cells. This particular type of communication has been implicated in several important biological processes such as cell growth and proliferation, embryogenesis as well as senescence, neurotransmission, hormonal secretion, tissue repair, leukocyte adhesion and inflammation, and oncogenesis [37-39].

Connexins, the elementary units which form gap junctions, are known to be expressed in fibroblasts. In these cells Cx43 is one of the most abundantly expressed connexins, nevertheless, its exact functional role remains to be fully elucidated. Direct cell-to-cell communication through Cx43 is thought to play a major role in wound healing. This is a dynamic process involving the upregulation of some of the known connexins such as Cx26 and Cx30, while Cx43 is strongly down-regulated. This phenomenon is linked to increased cell proliferation and migration of keratinocytes in the skin, thus suggesting an active role of this particular subunit in wound coverage and re-epithelization [19]. By using antisense oligodeoxynucleotide to Cx43 the same authors showed that artificially targeting Cx43 expression ultimately leads to an accelerated rate of the wound repair process. In addition, it has been recently demonstrated that in several cancer cell lines GJIC correlates exceptionally well with the rate of Cx43 expression. Cesen-Cummings et al. [40] demonstrated how lung cancer cell lines derived from mouse and humans lung carcinoma are characterized by low or absent levels of Cx43 expression when compared with the normal non-transformed counterpart. It is therefore appealing to correlate the reduced GJIC in lung fibroblasts derived from IPF patients with uncontrolled proliferation and ECM protein synthesis. Thus, we considered the possibility that behind the loss of proliferative control in IPF lays an altered function of the fibroblast-to-fibroblast communication mediated by connexins. We set out to determine whether an altered communication between fibroblasts might be implicated in IPF pathogenesis in an *in vitro *model. Indeed, we have previously shown that fibrotic fibroblasts possess distinct features that characterize their unique phenotype. Not only they are able to differentially drive inflammatory cell responses [41], but they also show reduced PgE2 synthesis and higher proliferation rates when compared to normal lung fibroblasts [42,43].

By using primary lung fibroblast cell cultures from healthy and IPF patients we demonstrate that Cx43 is reduced at the mRNA level in IPF-derived fibroblast lines. Furthermore, we assessed gap junctional activity with a dye-loading technique by means of flow cytometry [44], demonstrating that the altered transcription of connexin 43 has a functional counterpart in an abnormal reduced gap junctional intercellular communication in fibrotic fibroblasts. However the decrease of Cx43 mRNA is not associated to a parallel decrease of Cx43 protein level. Indeed, it is well-known that the amount of Cx43 expressed at the membrane level represents only a minor fraction of the total amount of the intracellular protein and that Cx43 turns over with a half-life of only between 1.5–5 h, even after incorporation into gap junctional plaques [45, 46]. This rapid rate of degradation has been observed in a wide variety of mammalian systems including primary and established tissue culture cells [47, 48], whole organs [49], and intact animals [50]. It has been shown that reducing connexin degradation with inhibitors of the proteasome is associated with a striking increase in gap junctional plaque assembly and intercellular dye transfer in cells [51]. It is possible that, in a non-trasformed cell type, such as the lung fibroblasts examined in the present study, a decrease of the rate of connexin transcription (mRNA levels) and protein synthesis, not accompanied by a parallel decrease of degradation, might reduce functional gap junction assembly more than the total cellular amount of Cx43. Further studies are necessary to support such hypothesis.

The technique used in the present study to assess GJIC utilizes donor cells as a source of the gap junction-permeable dye calcein for the recipient cells. Thus, the time course of the dye spreading depends not only on the intrinsic ability of recipient cells to allow the spreading of the dye, but also on the coupling of donor cells with recipient cells [52]. Ultimately, the time course depends on the number of active gap junctions and on their permeability for the selected dye. In some experiments, carbenoxolone, a specific gap junction blocker, was added to ascertain whether the intercellular transfer assay was effectively dependent on GJIC. Our results show that carbenoxolone abrogated calcein spreading into recipient cells, therefore confirming the reliability of our data on GJIC obtained by the dye-loading technique.

Our main experimental setup employed donor cells homologous to recipient cells. This condition simulates the cell-to-cell communication between phenotypically similar cells belonging to the same tissue or microscopic area, but adds nothing to the cell-to-cell communication taking place in the boundary between healthy and diseased section of the same organ. Similarly to our homologous GJIC experiments, heterologous GJIC was reduced in fibrotic fibroblasts even if the donor cells used were characterized by a normal gap junctional activity. In this regard Zhang et al. [53] who studied defective heterologous dye spreading between normal and cancer cell lines clearly demonstrated that reduced GJIC might explain the tumorigenic potential of a cell line only when it reaches a minimal threshold of Cx43 coupling. The alteration of the gap junctional activity between normal donor and diseased recipient cells might thus suggest that *in vivo *the altered cell-to-cell communication in fibrotic fibroblasts releases these cells from the control of the sourrounding normal areas. Under certain conditions, this is believed to be of paramount importance in the cascade of events that drive an altered cell type to escape from the restraints of contact-inhibition, thus facilitating uncontrolled cell proliferation.

In an experimental model of cisplatin induced damage, Cx43 expression and GJIC were found to be reduced in primary human lung fibroblasts [54]. Interestingly, p53, a well known lung cancer marker, was found to be elevated in conjunction with the aforementioned cell-to-cell alterations. Moreover, Cx43 deletion in mice results in a higher susceptibility to lung cancer [55}, while transfection of Cx43 in lung carcinoma cells expressing undetectable level of this protein inhibits cell growth and tumorigenicity in mice [56]. Recently, several studies have tried to correlate IPF with lung cancer with conflicting results [57, 58]. It would be tempting to hypothesize that the reduction of Cx43 and defective cell-to-cell communication we found in IPF fibroblasts is similar to what is described in cancer. This lends support to the idea that the uncontrolled proliferation of fibroblasts that characterize IPF may have some similarities to lung cancer.

Of course an *in vitro *model representing cell-to-cell communication cannot fully reflect the *in vivo *communication between cells in either normal or diseased lung. Further research is needed to unravel the steps influenced by gap junction dysfunction in IPF. However, this study proposes a novel pathogenetic alteration in primary lung fibroblasts from IPF/UIP patients: impairment of cell-to-cell communication. These phenomena may well explain some of the known pathological characteristic of this devastating disease that still represents a challenge to the medical treatment.
